# Examining the Process and Impact of Social Problem Solving in Autistic Children

**DOI:** 10.1007/s10803-024-06261-1

**Published:** 2024-02-23

**Authors:** Morgan L. McNair, Victoria Mondejar, Erin J. Libsack, Nicole H. Mordekai, Clark McKown, Nicole M. Russo-Ponsaran, Matthew D. Lerner

**Affiliations:** 1 Department of Psychology, Stony Brook University, Stony Brook, NY 11794-2500 USA; 2 American Medical Program at Tel Aviv University, Sackler School of Medicine, New York, NY 10065 USA; 3 Department of Psychiatry & Behavioral Sciences, Rush University Medical Center, Chicago, IL 60612 USA; 4 AJ Drexel Autism Institute, Drexel University, Philadelphia, PA USA

**Keywords:** Autism, Social cognition, Social problem solving, Social emotional learning

## Abstract

Social problem solving (SPS) represents a social cognitive reasoning process that gives way to behavior when individuals are navigating challenging social situations. Autistic individuals have been shown to struggle with specific aspects of SPS, which, in turn, has been related to social difficulties in children. However, no previous work has measured how SPS components not only relate to one another but also discretely and conjointly predict autism-related symptoms and social difficulties in autistic children, specifically. Fifty-eight autistic children (44 male; 6–10 years old, *M*_*age*_=8.67, *SD*_*age*_=1.31) completed a self-administered, computerized assessment of SPS. To elucidate how SPS components discretely, and combined, contribute to autism-related symptoms and social difficulties, commonality analyses were conducted for each measure assessing autism-related symptoms and social difficulties. Socially normative problem identification, goal preference, and solution preference were related to fewer parent-reported autism-related social difficulties. Measures related to autism symptomatology, social perspective taking, and emotion recognition were not significantly associated with discrete SPS components in this sample. The problem identification aspect of SPS contributed the most unique variance to parent-reported autism-related social difficulties, while shared variance across all SPS components accounted for substantial variance in both parent-reported autism-related social difficulties models. Results suggest that SPS components are interrelated, but distinct, constructs in the autistic population. These findings not only further clarify the impact of SPS components on autism-related symptoms and social difficulties, but also have implications for refining SPS-focused interventions in the autistic population.

**Supplementary Information:**

The online version contains supplementary material available at 10.1007/s10803-024-06261-1.

While autistic individuals^1^ are known to experience challenges in terms of social functioning (e.g., social behavior, emotion recognition, social perspective taking; Hobson, 2013), less is known about the specific cognitive processes involved in achieving – or failing to achieve – positive social functioning. Specifically, to engage in a social behavior successfully (e.g., initiate an interaction), an individual must encode and interpret the current situation, decide how to proceed, and enact a behavioral response – in other words, they engage in social problem solving (SPS; D’zurilla et al., 2004a; Lipton & Nowicki, 2009; McKown et al., 2009). Autistic people have been shown to demonstrate difficulties with SPS broadly (Channon et al., 2001; Goddard et al., 2007; Hochhauser et al., 2015). However, little is known about how the discrete components of SPS relate to social difficulties in autism, such as observed and task-measured social skills as well as autism spectrum disorder (ASD) symptomatology, in autistic children. Further, SPS components are intercorrelated; thus, efforts to examine their relationship to autism-related symptoms and social difficulties must prioritize disentangling their unique (i.e., each component) and common (i.e., general SPS ability) contribution to such relationships (McKown, 2019; McKown et al., 2013; Russo-Ponsaran et al., 2021). An investigation that accounts for both unique and common factors in specifying the impact of SPS on autism-related symptoms and social difficulties is needed to better refine formulations and assessment of SPS in this population.

## The Social-Emotional Learning Model and its SPS Components

There are several theoretical models of social perception and cognition that include SPS within their framework. A widely cited model of social cognition, the Social Information Processing Model (Crick & Dodge, 1994), outlines five steps children go through before making a behavioral response to a social situation: encoding, interpreting, establishing goals or desired outcome, constructing a response/solution, and making a response decision. Crucially, three of the social information processing steps – encoding (i.e., problem identification), establishing goals or desired outcome (i.e., goal preference), and constructing a response/solution (i.e., solution preference) map onto the construct of SPS used in this study. This model has been highly influential in shaping modern conceptions of social cognition and perception (e.g., Fite et al., 2008; Kupersmidt et al., 2011; Lemerise & Arsenio, 2000) but is limited by the complexity and challenges in simultaneous operationalization of each of the steps (Beauchamp & Anderson, 2010; Woods, 2010).

More recent models of social cognition have sought to offer a more streamlined and testable process (D’zurilla et al., 2004b; Lipton & Nowicki, 2009; McKown et al., 2009). Among the most influential contemporary empirical models is the Social-Emotional Learning Model (Lipton & Nowicki, 2009), which separates social-emotional learning into three domains: social awareness (the ability to identify and label emotions in others from nonverbal cues), social meaning (interpretation of the social problem), and social reasoning (the ability to judge the social situation and generate a behavioral response). This latter domain is also known as SPS but places it within a larger framework of social cognition.

Further, within this model, SPS comprises several discrete components: problem identification, goal selection, and solution selection. Though SPS components are often presented from problem identification to solution preference, the SPS process does not necessarily unfold in a fixed or linear sequence and often occurs out of conscious awareness (McKown et al., 2009). While theoretical models support the breakdown of SPS in this manner and prior research has demonstrated good internal consistency for measures of these SPS components independently (e.g., Maydeu-Olivares & D’Zurilla, 1996; McKown, 2019; McKown et al., 2013; McKown et al., 2016; Russo-Ponsaran et al., 2021), there is scarce research examining each SPS component conjointly within the same subjects. Specifically, little is known about whether SPS components are wholly discrete, represent largely shared variance (i.e., a general SPS cognitive function), or overlapping yet non-redundant constructs with adequate divergent and predictive validity (Russo-Ponsaran et al., 2021). Thus, efforts to understand performance on SPS components in populations with difficulties in social functioning requires a procedure and analysis that measures SPS components discretely.

## SPS in Autism

Research has shown autistic children demonstrate varying levels of difficulties with discrete SPS components, problem identification, interpretation, and solution preference and construction (e.g., Bottema-Beutel et al., 2019; Mazza et al., 2017). Many autistic children experience challenges with understanding social conflicts (Embregts & Van Nieuwenhuijzen, 2009; Mazza et al., 2017; Ziv et al., 2014), and these difficulties extend to interpreting social cues, such as the actions and words of others, and the intentions underlying social problems. Specifically, autistic children often attribute hostile intent to neutral, ambiguous, or unintentional social conflicts (Bottema-Beutel et al., 2019; Mazza et al., 2017; Ziv et al., 2014) and some autistic children judge others’ actions in social transgressions, whether the actions were intentionally harmful or not, more harshly than non-autistic individuals do (Rogé & Mullet, 2011). Harsher responses to unpleasant social interactions may be related to interpretation of intent or the tendency of autistic children to focus on negative and emotional information during social interactions (Embregts & Van Nieuwenhuijzen, 2009).

In autistic children and adolescents, challenges related to generating and selecting socially effective responses and solutions to social problems have been replicated (e.g., Channon et al., 2001; Russo-Ponsaran et al., 2018; Ziv et al., 2014). When asked to produce social problem solutions, autistic children generate fewer novel solutions than do non-autistic peers (Bernard-Opitz et al., 2001). Further, autistic children and adolescents have been shown to select more passive (Channon et al., 2001), avoidant (Ziv et al., 2014), and nonsocial solutions (Flood et al., 2011). Importantly, the SPS difficulties in autism experienced in childhood have been shown to persist into adulthood (e.g., Buon et al., 2013; Channon et al., 2014; Goddard et al., 2007), suggesting that better understanding and supporting SPS in autistic children may have implications across development. While autistic individuals demonstrate difficulties with various components of SPS, less work has measured multiple constructs of SPS within the same person (Russo-Ponsaran et al., 2018; Russo-Ponsaran et al., 2019).

## SPS and Autism-related Symptoms and Social Difficulties

In unpacking the role of social-emotional learning aspects in autism, it is important to assess how such aspects impact autism-related symptoms and social difficulties. More research has focused on the social awareness (e.g., Lozier et al., 2014; Trevisan & Birmingham, 2016; Uljarevic & Hamilton, 2013) and social meaning components of social-emotional learning (i.e., theory of mind; Happé & Frith, 1995; Tager-Flusberg, 2007; Velikonja et al., 2019) than social reasoning (e.g., Buon et al., 2013; Gómez-Pérez et al., 2019), with the relationship between SPS and autism-related symptoms and social difficulties being particularly understudied. In both autistic and non-autistic school-aged children, greater social awareness skills have been associated with better problem identification (Russo-Ponsaran et al., 2015). Additionally, autistic children with emotion recognition difficulties perform worse on identification of social problems and interpretation of intent (Meyer et al., 2006). Conversely, in this same study, autistic children who demonstrated more parent-reported prosocial and empathetic behaviors were more likely to respond to hypothetical social transgressions in a less aggressive manner (Meyer et al., 2006).

## The Present Study

The present study evaluated how performance on discrete components of SPS, specifically problem identification, goal preference, and solution preference, predict social difficulties in autistic children. Given the complexities of SPS, the present study sought to further breakdown these relationships to elucidate how SPS components discretely, and combined, contribute to autism-related symptoms and social difficulties. Thus, it was hypothesized that (1) autistic children who engage in more normative *social problem identification* as well as more normative *social problem goal* and *solution preferences* would demonstrate fewer autism-related symptoms and social difficulties. It was also hypothesized that (2) the shared variance among all SPS components would account for the greatest variance in autism-related symptoms and social difficulties across multiple measurement approaches (e.g., questionnaire and task-based assessments). Beyond the shared variance among SPS components, this study explored the relative unique and shared contributions of discrete SPS components to autism-related symptoms and social difficulties variance.

## Method

### Participants

Participants were 57 autistic children ages 6–10 years (44 male; *M*_*age*_=8.67, *SD*_*age*_=1.31). All children had ASD diagnoses confirmed with the Autism Diagnostic Observation Schedule, 2nd Edition (ADOS-2; Lord et al., 2012), using the ADOS-2 recommended cutoffs and conducted by a research-reliable administrator. This included masters-level staff, doctoral candidates, and doctorate-level faculty. A cognitive assessment using the Kaufman Brief Intelligence Test, 2nd Edition (KBIT-2; Kaufman & Kaufman, 2004) was also completed for each participant. Participants were recruited from research registries and with flyers distributed at local schools and community agencies. Parents/caregivers completed a screening questionnaire (SCQ; Social Communication Questionnaire, Rutter et al., 2003) to determine if the child met the cutoff for elevated ASD symptomatology. Exclusion criteria included IQ < 80 as measured by the KBIT and failure to meet ASD criteria on the SCQ and ADOS-2. Fifty-nine participants met these criteria; however, one participant did not complete the SPS measures and another participant dropped out of the study, resulting in a sample of 57 participants. See the demographic table (Table 1) for the age, sex, IQ, and race and ethnicity distribution of the sample. All study procedures were approved by and conducted in accordance with the ethical standards of the Institutional Review Boards of both the data collection and housing sites as well as the 1964 Helsinki Declaration and its later amendments or comparable ethical standards.

**Table 1:** Demographic Information

Demographic Variable	*n* (% *N* = 57)	M (SD)	Range
Age		8.59 (1.32)	6.02–10.97
IQ^a^		104.11 (14.15)	80–133
SSIS^b^		73.51 (12.47)	48–109
SRS-2^c^		77.68 (9.79)	51–90
ADOS-2 CSS^d^		7.47 (1.91)	4–10
SELweb SPT^e^		87 (16.8)	56.33–121.30
SELweb ER^f^		98.67 (12.03)	66.62–121.46
SELweb SPS^g^		92.20 (20.40)	30.06–115.13
SELweb SPS Problem ID^h^		0.92 (0.19)	0.17–1.00
SELweb SPS Goal^i^		92.28 (20.65)	16.40–112.04
SELweb SPS Solution^j^		95.48 (17.38)	37.70–116.91
Sex			
Female	13 (22.8%)		
Male	44 (77.2%)		
Race/Ethnic Background			
White/Caucasian	52 (91.2%)		
Black/African American	1 (1.8%)		
Asian/Asian American	4 (7%)		
Native American/American Indian or Alaskan Native	0 (0%)		
Native Hawaiian or Other Pacific Islander	0 (0%)		
Not Listed	1 (1.8%)		
Not Disclosed	2 (3.5%)		
Hispanic/Latino	7 (12.3%)		

*Note. *^a^Full Scale IQ measured with the KBIT-2; ^b^SSIS standard score; ^c^SRS-2 Total T-score; ^d^ADOS-2 Comparison Severity Score; ^e^SELweb Social Perspective Taking Module standard score; ^f^Emotion Recognition Module standard score; ^g^SELweb Social Problem-Solving Module standard score; ^h^Normative Problem Identification Selection Ratio – A standard score has not been produced for the Problem Identification component of the SELweb SPS Module; ^i^SELweb Social Problem-Solving Goal Preference standard score; ^j^SELweb Social Problem-Solving Solution Preference standard score

### Procedure

All data used in the present study were drawn from a larger study evaluating the usability as well as reliability and validity of SELweb in a sample of autistic children. After screening, eligibility was determined via the SCQ (scores ≥ 11; Norris & Lecavalier, 2010), 104 children were invited to complete further testing. Of those 104 children, 92 participants and their parents provided informed assent and consent, respectively. Of those 92 participants, 57 met inclusion criteria for full participation in the study and completed the relevant measures. Participants completed all experimental components, lasting approximately three hours, over the course of one or two sessions either at Stony Brook University or Rush University Medical Center. Parents escorted participants to the research visit(s) but did not stay with participants during testing. All aspects of the study, other than the ADOS-2, were administered by trained research staff and undergraduate research assistants. For the SELweb administration, participants were seated at a computer across from a research assistant.

### Measures

#### SELweb Assessment (McKown et al., 2016; Russo-Ponsaran et al., 2019)

SELweb Early Elementary (EE), is a computerized, web-based assessment, with built-in narration for each module, that evaluates various domains of social-emotional learning in children, including social awareness, social reasoning, social meaning, and self-control. Participants are presented with five modules: facial emotion recognition (social awareness), social problem solving (social reasoning), social perspective taking (social meaning), delay of gratification, and frustration tolerance (self-control). The assessment takes approximately 35 min to complete. SELweb EE is designed and normed for students in K-3rd grade, and the sample of the current study ranges from 1st to 5th grade (*M* = 3rd grade, *SD* = 1.22 grades). While SELweb EE was designed and normed for children K-3rd grade, research examining the usability, reliability, and validity of SELweb in autistic children with IQ ≥ 80 (Russo-Ponsaran et al., 2019) extended the age range of this measure to 10 years old (~ 5th grade) for this population; this extension was made given the developmental delays and extant social-emotional challenges associated with autism. Raw scores from each SELweb module have been normed on large samples of typically-developing children (e.g., 4,462 children; McKown et al., 2016) and converted to standard scores (M = 100, SD = 15; Russo-Ponsaran et al., 2019). Further, all SELweb tasks have been well validated. Convergent validity has been largely supported, with medium to large associations (*r* coefficients 0.37 − 0.88) and with convergent individual measures as well as modeled latent constructs (e.g., McKown, 2015, 2019; McKown et al., 2013). Discriminant validity was similarly established regarding non-convergent measures (McKown, 2015). Additionally, past research has supported the construct discrimination among social emotional learning components, both at measured and latent levels of analysis. Internal consistency has likewise been demonstrated to be adequate (Cronbach’s alphas > 0.73-0.93; McKown, 2019; Russo‐Ponsaran et al., 2019).

For the purposes of this study, we examined the SPS, social perspective taking, and facial emotion recognition modules. The social perspective taking and emotion recognition standard scores were used. Analysis of SELweb SPS module responses did not use standard scores; responses were re-coded based on the type of response selected.

#### SELweb SPS Module

**Fig. 1:**
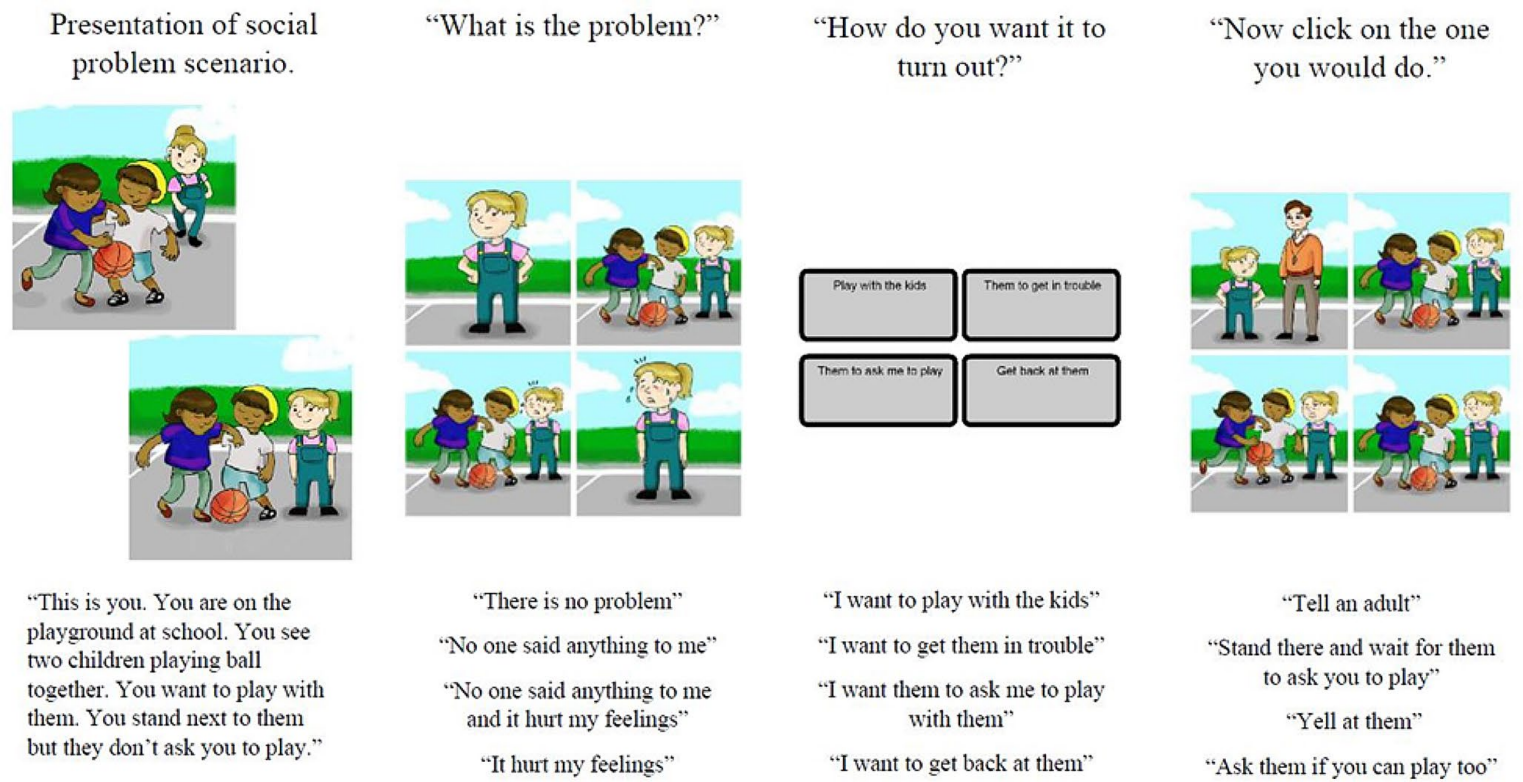
Schematic of SELweb social problem solving (SPS) module

Participants were presented with six illustrated and narrated vignettes of social problems. In the SPS module (Fig. 1), vignettes were posed from a first-person perspective, and after each vignette, participants were asked a series of questions about the social problem, including *problem identification* (“What is the problem?”), *goal preference* (“How do you want it to turn out?”), and *solution preference* (“Now click on the one you would do.”). Response options for each question were presented in multiple-choice format. If the participant needed to hear the response option again, they could click on the option, and it would be read aloud. Once a response option was selected, the paradigm would move on to the next question. In this sample of autistic children, Cronbach’s α for the overall average score on the SELweb SPS module was 0.89 (Russo-Ponsaran et al., 2019).

For the purposes of this study, each SPS component was recoded to categorize *socially normative* and *non-normative* response selections. For *problem identification*, response options to “What is the problem?” included a physical problem (e.g., “No one said anything to me”), a feelings-related problem (e.g., “It hurt my feelings”), a physical and feelings-related problem (e.g., “No one said anything to me and it hurt my feelings”), and no problem (e.g., “There is no problem”). All selections but ‘no problem’ were considered *socially normative* selections and delineated as 1’s (a *non- normative* identification selection was coded as 0). *Problem identification* scores (1 or 0) for each SPS vignette were summed (highest total possible was 6, meaning all *problem identifications* were socially normative) and then turned into a percent of normative *problem identification* selections (*problem identification* score divided by 6).

For *goal preference*, response options to “How do you want it to turn out?” included a prosocial (e.g., “I want to play with the kids”), a problem-focused (e.g., “I want them to ask me to play”), a revenge (e.g., “I want to get back at them”), and a retributive preference (e.g., “I want to get them in trouble”). Prosocial and problem-focused preferences were coded as *socially normative* and delineated as 1’s; retributive and passive-aggressive preferences were coded as *socially non-normative* and delineated as 0’s. *Goal preference* scores (1 or 0) for each SPS vignette were summed (highest total possible was 6, meaning all *goal preferences* were *socially normative*) and then turned into a percent of normative *goal preferences* (*goal preference* score divided by 6).

For *solution preference*, response options to “Now click on the one you would do” included a normative (e.g., “Ask them if you can play too”), an authoritative (e.g., “Tell an adult”), a passive-avoidant (e.g., “Stand there and wait for them to ask you to play”), and an aggressive preference (e.g., “Yell at them”). All options but ‘normative’ were coded as *socially non-normative* and delineated as 0’s (selecting the *normative solution* choice was coded as 1). *Solution preference* scores (1 or 0) for each SPS vignette were summed (highest total possible was 6, meaning all *solution preferences* were socially normative) and then turned into a percent of normative *solution preferences* (*solution preference* score divided by 6).

#### SELweb Social Perspective-Taking Module

The social perspective taking (SPT) module included twelve animated vignettes. Participants were asked to identify the mental or emotional state of a character in each vignette from response options presented on the screen (e.g., identifying why a character said what they said). In this sample of autistic children, Cronbach’s α for the summed social perspective taking scores was 0.81 (Russo-Ponsaran et al., 2019).

#### SELweb Emotion Recognition Module

In the emotion recognition (ER) module, participants viewed 40 different faces of children and were asked to identify which emotion the face portrayed: Happy, Sad, Angry, Scared, or Just Okay. The Cronbach’s α for the emotion recognition module in this sample of children was 0.90 (Russo-Ponsaran et al., 2019).

#### Kaufman Brief Intelligence Test, 2nd Edition (KBIT-2; Kaufman & Kaufman, 2004)

KBIT-2 is a measure of cognitive ability for individuals between ages 4 and 90 years. The test, administered by a trained clinician or research assistant, produces standard scores, including crystalized (verbal) and fluid (nonverbal) IQs, and has been widely used in studies of autistic children (e.g., Granieri et al., 2020; Russo-Ponsaran et al., 2019).

#### Autism Diagnostic Observation Schedule, 2nd Edition (ADOS-2; Lord et al., 2012)

The ADOS-2 is considered a gold-standard diagnostic tool for characterizing ASD symptomatology. Administered by a trained, research-reliable administrator (i.e., masters-level staff, doctoral candidates, doctorate-level faculty), the ADOS-2 involves a series of interactive activities that include “social presses” intended to elicit normative social responses. For the present study, ADOS-2 Comparison Severity Score (CSS; Gotham et al., 2009), which quantifies autism symptom severity, was used as a measure to assess autism symptomatology.

#### Social Responsiveness Scale, 2nd Edition (SRS-2; Constantino, 2012)

The SRS-2, a 65-item parent- or caregiver-report questionnaire, assesses ASD symptomatology and autism-specific social impairment. SRS-2 items cluster into two overarching domains, Social Communication Interaction and Restricted Interest and Repetitive Behavior. Total SRS-2 raw scores range from 0 to 195, which are then converted into *T*-scores based on chronological age and biological sex. Higher *T*-scores indicate more ASD symptoms and social impairment. SRS-2 Total *T*-Score was used as a measure to assess autism-related social difficulties.

#### Social Skills Improvement System Rating Scale (SSIS; Gresham & Elliott, 2007)

The SSIS parent-report questionnaire is used to assess social skills, problem behaviors, and academic competence. The SSIS Composite Score (i.e., overall social skills) is converted to an age-based and gender-normed standard score. Correlations between subdomains and domains ranged from moderate to high (0.70 to 0.80), and the SSIS has been widely used in autism studies (e.g., Hill et al., 2017; Jamison & Schuttler, 2015). For this study, SSIS Standard Score was used as a measure to assess autism-related social difficulties.

### Data Analytic Plan

Frequency of responses for each SPS component were examined. Given the ordinal quality of SPS variables, nonparametric (Spearman’s) correlations were conducted between SPS components to evaluate the degree of convergence (and divergence) among them in autistic children. Correlations were conducted using SPSS Version 26. Further, the magnitudes of correlation coefficients for each SPS component pairing (i.e., problem identification and goal preference, problem identification and solution preference, goal preference and solution preference) were examined using Fisher’s *R*-to-*Z* transformation.

To test Hypothesis 1, that autistic children who engaged in better *social problem identification* as well as normative social problem *goal* and *solution preference* would present with fewer autism-related symptoms and social difficulties, Spearman’s correlations were conducted between performance on each discrete SPS component (*social problem identification*, *goal preference*, *solution preference*) and each measure of autism-related symptoms and social difficulties (SSIS standard score, SRS-2 Total T-score, ADOS-2 CSS, social perspective taking standard score, emotion recognition standard score). To test Hypothesis 2, that the shared variances among all discrete SPS components would account for the greatest variance in each measure of social difficulties in autistic children, commonality analyses (Nimon et al., 2008) were run with performance on discrete SPS components predicting each measure of autism-related symptoms and social difficulties (SISS and SRS-2). These models were also examined with respect to the exploratory component of Hypothesis 2, which sought to determine the relative contribution of the unique variance of discrete SPS components to autism-related symptoms and social difficulties. Post-hoc analyses were conducted for each measure of autism-related symptoms and social difficulties to include age and IQ as covariates. Commonality analyses were run using R 4.2.3 and RStudio.

Like a regression model, a commonality analysis provides the explained variance for each predictor of the outcome variable. However, commonality analysis further parses out explained variance into the unique effects of each predictor as well as the shared effects of each possible combination of predictors. Commonality analysis allows for a more nuanced untangling of how SPS relates to autism-related symptoms and social difficulties in this population.

## Results

For the SPS components of *problem identification* and *goal preference*, the majority of participants (> 62%) always (i.e., for all six SPS scenarios) provided socially normative responses (Table 2). For the SPS component of *solution preference*, socially normative responses across scenarios were more broadly distributed.

**Table 2:** Frequencies of Normative Responses by SPS Component and Bivariate Correlations between SPS Components

	Prob ID^a^	Goal^b^	Solution^c^
Scenarios with Socially Normative Responses	n (%)	n (%)	n (%)
0/6	0 (0)	4 (6.9)	11 (19.0)
1/6	1 (1.7)	2 (3.4)	3 (5.2)
2/6	2 (3.4)	1 (1.7)	4 (6.9)
3/6	2 (3.4)	3 (5.2)	12 (20.7)
4/6	1 (1.7)	4 (6.9)	9 (15.5)
5/6	6 (10.3)	7 (12.1)	5 (8.6)
6/6	45 (77.6)	36 (62.1)	13 (22.4)
	Prob ID	Goal	Solution
Prob ID	1	0.39**	0.49**
Goal		1	0.64**
Solution			1

*Note.* **p < .01; ^a^Prob ID = Normative Problem Identification Selection Ratio; ^b^Goal = Normative Goal Preference Ratio; ^c^Solution = Normative Solution Preference Ratio

Each SPS component (*problem identification*, *goal preference*, *solution preference*) was correlated with one another (all *p*s < 0.01; Table 2). Correlation coefficients ranged from medium to large (Cohen, 1992). Fisher’s *R*-to-*Z* transformation analyses revealed that the difference in correlation magnitudes was not significant between the problem identification–goal preference relationship and the problem identification–solution preference relationship (*z*=-0.66, *p* > .05). The difference in correlation coefficient were significant between the problem identification–goal preference relationship and the goal preference–solution preference relationship (*z*=-2.93, *p* < .005) as well as the problem identification–solution preference relationship and the goal preference–solution preference relationship (*z*=-2.30, *p* < .05).

### SPS Performance and Autism-related Symptoms and Social Difficulties

Socially normative *problem identification* and *goal preference* were associated with greater parent-reported social skills (SSIS; Table 3). Socially normative *problem identification* and *solution preference* were associated with fewer parent-reported autism-related social symptoms (SRS-2; Table 3). However, socially normative performance on discrete SPS components was not significantly associated with ASD symptomatology (ADOS-2 CSS) nor performance on the social perspective taking module (social awareness) and emotion recognition module (social meaning).

**Table 3:** Spearman’s Rho Correlations between Measures of Social Competencies and Performance on SPS Components

	Prob ID^a^	Goal^b^	Solution^c^
SSIS^d^	0.35**	0.27*	0.26^†^
SRS-2^e^	− 0.37**	− 0.24	− 0.27*
ADOS-2 CSS^f^	− 0.18	− 0.30	− 0.24
SPT^g^	0.11	0.17	0.22
ER^h^	0.09	0.15	0.13
Age	0.19	0.11	0.08
IQ^i^	0.12	0.07	0.19

*Note. *^†^*p* < .10, *p < .05, **p < .01; ^a^Prob ID = Normative Problem Identification Selection Ratio; ^b^Goal = Normative Goal Preference Ratio; ^c^Solution = Normative Solution Preference Ratio; ^d^ SSIS = Social Skills Improvement System Rating Scale standard score; ^e^SRS-2 = Social Responsiveness Scale, 2nd Edition, Total T-score; ^f^ADOS-2 CSS = Autism Diagnostic Observation Schedule, 2nd Edition, Comparison Severity Score; ^g^SPT = SELweb Social Perspective Taking Module standard score; ^h^ER = SELweb Emotion Recognition Module standard score

### Commonality Analysis

In both the SSIS and SRS-2 models, socially normative *problem identification* accounted for the most unique variance. Shared variance across SPS components accounted for the most common variance in each model (Table 4).

**Table 4:** Social competencies commonality analyses

SSIS^e^ Predictors (R^2^ = 0.11)	Prob ID^a^	Goal^b^	Solution^c^	%Total^d^
**Unique to Prob ID**	0.0395			36.20
Unique to Goal		0.0035		3.23
Unique to Solution			0.0042	3.88
Common to Prob ID & Goal	0.0031	0.0031		2.88
Common to Prob ID & Solution	0.0108		0.0108	9.89
Common to Goal & Solution		0.0137	0. 0137	12.58
**Common to all three predictors**	**0.0342**	**0.0342**	**0.0342**	**31.32**
SRS-2^f^ Predictors(R^2^ = 0.13)	Prob ID	Goal	Solution	%Total
**Unique to Prob ID**	**0.0507**			**38.16**
Unique to Goal		0.0030		2.26
Unique to Solution			0.0058	4.34
Common to Prob ID & Goal	0.0034	0.0034		2.56
Common to Prob ID & Solution	0.0142		0.0142	10.66
Common to Goal & Solution		0.0151	0.0151	11.35
**Common to all three predictors**	**0.0408**	**0.0408**	**0.0408**	**30.66**

*Note*. ^a^Prob ID = Normative Problem Identification Selection Ratio; ^b^Goal = Normative Goal Preference Ratio; ^c^Solution = Normative Solution Preference Ratio; ^d^% Total refers to the percent of variance the combination of predictors, or each predictor uniquely, contributes to each social competency model; ^e^SSIS = Social Skills Improvement System Rating Scale standard score; ^f^SRS-2 = Social Responsiveness Scale, 2^nd^ Edition, Total T-score; Bolded values indicate which SPS component(s), individually or combined, contribute the most variance within each model

Values in 2^nd^ through 4^th^ columns denote commonality coefficients, which represent the isolated, unique, and common variance each predictor (and combination of predictors) contributes to the dependent variable

### Post-hoc Analyses

Because age and IQ are likely to influence performance on SPS components and measures of autism-related symptoms and social difficulties, models were rerun with age and IQ included as covariates. In the SSIS model, age and socially normative *problem identification* accounted for the most unique variances, respectively, while shared variance across SPS components accounted for the most common variance (Supplementary Table 1). In the SRS-2 model, socially normative *problem identification* accounted for the most unique variance, while shared variance across SPS components accounted for the most common variance (Supplementary Table 2). Additionally, even though ADOS-2 CSS was not significantly associated with performance on discrete SPS components (Table 3), given that the ADOS-2 plays a critical role in receiving an ASD diagnosis, a supplemental commonality analysis was conducted to evaluate how performance on discrete SPS components predicts autism symptomatology (see Supplementary Materials).

## Discussion

This study measured discrete SPS components and examined how the unique and conjoint variance of performance on SPS components relates to autism-related symptoms and social difficulties in autistic children. Socially normative performance on discrete SPS components was related to fewer parent-reported autism-related social difficulties. *Problem identification* contributed most unique variance to parent-reported autism-related social difficulties, and shared variance across all SPS components accounted for the most common variance in both parent-reported autism-related social difficulties models, even after accounting for age and cognitive ability. Results suggest, while overlapping, SPS components can be seen as distinct, which is consistent with what is seen in non-autistic samples (e.g., Russo-Ponsaran et al., 2021) and supports their continued examination.

While performance on all SPS components correlated with each other, the strength of each relationships varied widely. The correlation coefficients of the problem identification–goal preference relationship and the problem identification–solution preference relationship were not significantly different from each other, while the relationship between *goal preference* and *solution preference* was significantly larger than the aforementioned relationships. Importantly, the association between *goal preference* and *solution preference* was the only large correlation, suggesting autistic children who select more socially normative goals for how they want a social problem to work out also select more socially normative solutions to social problems: an effective goal seems to beget an effective solution. Conversely, the smaller correlations were between *problem identification* and *goal preference* as well as *problem identification* and *solution preference*, suggesting that, in autistic children, being able to accurately identify the presence of a given social problem does not reliably relate to the selection of a socially normative goal or solution (Bauminger, 2002; Bottema-Beutel et al., 2019). That being said, initial identification of a social problem (i.e., *problem identification*) appears to be highly valuable for autistic children in navigating SPS. While moderate in effect, accurate social *problem identification* was significantly associated with socially normative *goal preference* and *solution preference*. Further, as shown via the commonality analysis findings, being able to identify neuro-normative *goal* and *solution preferences* does not necessarily relate as much to parent-reported social skills as *problem identification* ability. In contrast to previous studies evaluating the discriminant validity of SPS components (D’Zurilla & Maydeu-Olivares, 1995; Maydeu-Olivares & D’Zurilla, 1996), this variation in association strength suggests that discrete SPS components are overlapping, but non-redundant, in their measurement. The findings also suggest such construct discrimination may uniquely present in autistic children, or that the SELweb SPS module may be particularly adept at establishing such differences. Further, the findings provide important guidance for which SPS aspects should be examined to best understand how autistic children engage in SPS. The relationships between SPS components further highlight that, while SPS may reflect a latent cognitive construct, observed differences in performance across components are measurable, such that their utility and nosology may be investigated in autistic children.

Contrary to our hypothesis, socially normative performance on SPS components was only related to parent-reported autism-related social difficulties, specifically fewer parent-reported autism-related social difficulties (SSIS/SRS). This suggests that behaviors of autistic children who engage in adaptive internal SPS – at any step – manifest in ways that appear functional and prosocial to their parents, and these relationships are consistent with prior literature examining SPS abilities and social functioning in autism (Jackson & Dritschel, 2016; Meyer et al., 2006). Despite sparse research surrounding the relationship between SPS performance and autism-related symptoms and social difficulties, literature on interventions that address SPS skills reports autistic individuals can make notable gains in SPS abilities (e.g., Bauminger, 2002; Bernard-Opitz et al., 2001; Boujarwah et al., 2010; Pugliese & White, 2014). However, SPS gains do not reliably translate into observed behavior, suggesting an important gap remains between change in SPS and change in parent-observed behavior. Thus, the findings provide a promising groundwork for future research to evaluate the impact of SPS interventions on changes in autism-related symptoms and social difficulties as well as development of SPS skills in autistic children.

The hypothesis that shared variance between SPS components (i.e., a pseudo-latent SPS capacity) would predict the most variance in autism-related symptoms and social difficulties was partially supported, with several unexpected findings for discrete SPS components. The use of commonality analysis permitted a more nuanced examination of the contribution of the unique effects of each predictor as well as the shared effects (i.e., common variance) of each possible combination of predictors (Nimon et al., 2008), which has been rarely used in the autism literature (Santore et al., 2020). *Problem identification* contributed the most unique variance for both parent-reported autism-related social difficulties measures – equal to and greater than the shared variance across SPS components in the SSIS and SRS-2 models, respectively. While *problem identification* was the SPS component that contributed the most unique variance to parent-reported autism-related social difficulties, chronological age, when included as a covariate in the SSIS model, also accounted for nearly the same amount of unique variance as *problem identification*. The SSIS is normed both by sex and age, which may explain this effect. Although, this effect of age is contradictory to Bailey and Im-Bolter (2020)’s finding that *problem identification* scores in a non-autistic population did not differ by age (7- and 8-year-olds vs. 11- and 12-year-olds), which suggests that a more in-depth evaluation of the relationship between age and *problem identification* performance in autistic children, specifically, may be warranted. That being said, the present study’s findings still suggest *problem identification* seems to be a uniquely important SPS component for autistic children in achieving positive social functioning – in other words, simply *identifying that a problem exists* may be enough for many autistic children to achieve positive social functioning (e.g., McAfee, 2002). It may be that identifying the presence of a social problem allows for autistic children to begin the SPS navigation process, catalyzing use of additional social cognitive strategies to navigate subsequent SPS components, such as goal and solution preference. However, a failure to identify that a social problem is present may short-circuit the SPS navigation process (i.e., SPS goal and solution preferences may not feel applicable, necessary, or appropriate to the individual if a social problem does not exist), resulting in more social difficulties (e.g., the social problem appears to be ignored). In this way, these findings align with the social information processing speed model of social functioning in autism (Keifer et al., 2020; Mendelson et al., 2016), which suggests that how quickly an *initial social processing step* is achieved (the proverbial “foot in the door”) is more important than the invocation of subsequent steps in yielding positive social outcomes for autistic children. Additionally, as discussed in Bailey and Im-Bolter (2020), adults often assist children with interpersonal conflict intervention by asking about the nature of the conflict at hand (and instruct children to share or take turns - a common and widely applicable solution). Consequently, from a young age, a child’s attention in an SPS-based situation is frequently directed toward *problem identification*, which is an SPS component considered highly ingrained and an easier aspect of the SPS process for non-autistic youth (Bailey & Im-Bolter, 2020). While social skill interventions for non-autistic youth may focus more on more difficult aspects of SPS, such as strategy and solution evaluation, a focus on *problem identification* may be more applicable for autistic youth in this age range. Taken together, the findings suggest *problem identification* may serve as a key focus for SPS and other social cognition-focused interventions.

Importantly, shared variance across all SPS components *did* account for the most common variance in each model of autism-related symptoms and social difficulties, and a considerable portion of the total variance in each. These findings support Hypothesis 2, suggesting a “pseudo-latent” SPS capacity may exist, and undergird social cognitive processing. Future research should examine this possibility and the possibility that it may also reflect a more generalized neurocognitive processing capacity (Lerner et al., 2015).

### Clinical Implications

Findings from the present study have several clinical implications for the autism field. Numerous interventions for SPS skills in autism already break down SPS training into its discrete components (e.g., Bonete et al., 2015; Cote et al., 2014; Solomon et al., 2004; Stichter et al., 2010). With a better understanding of how performance on specific SPS components predict autism-related symptoms and social difficulties, interventions can be tailored to the specific needs of an autistic individual rather than - potentially unnecessarily - training all SPS components. The breakdown of SPS into discrete components also aligns with the Distillation and Matching Model framework of intervention (Chorpita et al., 2005). This modular approach to SPS intervention provides individualized treatment planning and may be implemented as a low-intensity intervention (Libsack et al., 2022), thus decreasing the time-intensive and financial burden of interventions on autistic people and their families.

The relationship between SPS abilities and autism-related symptoms and social difficulties may also have implications for how autistic children make and maintain friendships. Autistic children experience comparable levels of conflict with friends as non-autistic children (Mendelson et al., 2016). Friendship conflicts are a normative process, and the conflict-resolution experience teaches children crucial skills regarding how to navigate and strengthen friendships. However, difficulties with SPS abilities, compounded with similar amount of friendship conflicts, could impact the learning experienced during friendship conflict-resolutions for autistic children. Correctly identifying the presence of a social problem was related to fewer autism-related symptoms and social difficulties; thus, targeting problem identification in social skill interventions may aid autistic children in the conflict-resolution experience.

### Limitations & Future Directions

Several limitations exist with respect to the generalization of results of this study and are worth noting. First, the present study offered a moderate sample size. However, a larger sample size would allow for narrower confidence intervals and reduce sampling error. Second, there was limited variation in race, ethnicity, and sex. Specifically, the sample consisted of predominantly White, male children with cognitive abilities in the average to above average range. Future research should seek to replicate this work with a more diverse sample in race, ethnicity, and sex and also in autistic children with lower cognitive abilities. The results of the present study may differ as a result of more diverse samples at different ages (i.e., young children, adolescents), allowing for greater generalizability of findings. Further, future studies will need to consider the receptive language demands of the current study’s SPS task when investigating SPS in autistic children with lower cognitive abilities. Third, the SELweb SPS module had a fixed and finite number of items and scenarios to assess SPS; thus, while SELweb was normed on a large non-autistic population, the range of potential responses (and variance) among individuals was somewhat low. Such analyses and SPS component coding mechanisms should also be considered with other SPS measures designed to capture a broader range of scenarios and ages. Fourth, the current study did not include a comparison group. However, the module has been tested on a large group of children and SPS scores have been standardized (McKown et al., 2016; Russo-Ponsaran et al., 2019), which allowed for comparisons against this larger sample. Nonetheless, including a comparison group in future studies of this sort would provide insight into how performance on discrete SPS components relates to social competencies in non-autistic children and a more nuanced lens of potential SPS differences between non-autistic and autistic children.

Two of the measures used to evaluate autism-related symptoms and social difficulties in the sample were completed via parent-report, which has been shown to be associated with informant biases and common method variance concerns (Bank et al., 1990; Valentiner & Mounts, 2017). While these confounds bear note, SELweb modules measure child performance; thus, if parents are systematically detecting an epiphenomenon of the construct of interest (i.e., autism-related symptoms and social difficulties), they appear to be doing so with sufficient consistency to detect effects, and it is valuable to capture these effects, even in the face of potential bias. Moreover, post-hoc analyses indicate that at least two plausible sources of potential bias, child age and IQ, do not substantially attenuate or explain the relation between SPS components and autism-related symptoms and social difficulties, further ameliorating this concern. However, future work should continue to identify other variables that may explain the large portions of residual variance in these outcomes. Further, the study did not include teacher-report of autism-related symptoms and social difficulties, which would have been valuable given that teachers observe much of a child’s social interactions throughout the day. Recent work has demonstrated great clinical utility in assessing functional impairment of autistic children through discrepancies between parent- and teacher-report of ASD symptom severity (Lerner et al., 2017); thus, future replications of the present study should include reports from both informants, as well as other measures of social cognitive factors, to capture a more comprehensive perspective of autism-related symptoms and social difficulties.

Additionally, a burgeoning area of literature in the autism field has focused on compensation (e.g., Corbett et al., 2021; Livingston et al., 2019a, b) and passing as non-autistic (i.e., Libsack et al., 2021). One possibility is that some autistic children who better identify problems may also be better at masking, thus presenting with fewer parent-reported autism-related social difficulties. Future research should examine how masking and passing as non-autistic map onto SPS aspects in autistic children. Lastly, the social motivation hypothesis of autism suggests that, from an early age, autistic individuals attend less to social stimuli, such as faces and eye-contact, than non-autistic individuals because they find it less rewarding (Chevallier et al., 2012), resulting in decreased opportunities to engage with and learn from social stimuli and subsequent difficulties in social skill development. Given prior literature on how autistic individuals process social and nonsocial rewards and the downstream effects such processing has on social skill development (Clements et al., 2018), future studies should evaluate the role social motivation may play in how autistic children navigate SPS.

## Conclusion

This study examined several SPS components in autistic children, revealing within- and between-person variation and suggesting each component represents discrete but overlapping social-cognitive constructs in this population. Further, while performance on each SPS component was associated with parent-reported autism-related social difficulties, results suggest problem identification may be the most impactful individual SPS component for yielding positive parent-reported autism-related social difficulties. Understanding how performance on discrete SPS components relates to how autistic individuals present and function socially not only has implications for social interventions in the autistic population but also underscores the potential downstream impact SPS has on the social development of autistic children.

## Electronic Supplementary Material

Below is the link to the electronic supplementary material.

Supplementary Material 1

## Footnotes

1 As recommended by both autistic self-advocates and autism researchers (e.g., Bottema-Beutel et al., 2021), identity-first language/“on the autism spectrum” will be used throughout the manuscript.

## Declarations

Earlier iterations of this work were presented at the 2021 International Society for Autism Research Conference.
