# Supplementary material for: Examining the Process and Impact of Social Problem Solving in Autistic Children
Source: J Autism Dev Disord. 2024 Feb 23;55(3):789–802. doi: 10.1007/s10803-024-06261-1 (PMC11828759; doi:10.1007/s10803-024-06261-1)
Supplement: Supplementary file 1 — Supplementary Material 1 [file 10803_2024_6261_MOESM1_ESM.pdf]

Supplemental Table 1. *SSIS Commonality Analyses with Age and IQ as Covariates*

**SSIS[g] Predictors** (R^2=.14)	**Prob ID[a]**	**Goal[b]**	**Solution[c]**	**IQ[d]**	**Age[e]**	**%Total[f]**
Unique to Prob ID	***0.0306***					***21.28***
Unique to Goal		0.0044				3.03
Unique to Solution			0.0036			2.49
Unique to IQ				0.0010		0.68
Unique to Age					***0.0340***	***23.69***
Common to Prob ID & Goal	0.0029	0.0029				1.99
Common to Prob ID & Solution	0.0088		0.0088			6.15
Common to Goal & Solution		0.0142	0. 0142			9.87
Common to Prob ID & IQ	0.0013			0.0013		0.88
Common to Goal & IQ		0.0004		0.0004		0.29
Common to Solution & IQ			-0.0006	-0.0006		-0.44
Common to Prob ID & Age	0.0080				0.0080	5.59
Common to Goal & Age		-0.0011			-0.0011	-0.75
Common to Solution & Age			0.0010		0.0010	0.73
Common to IQ & Age				-0.0006	-0.0006	-0.40
Common to Prob ID, Goal, & Solution	***0.0308***	***0.0308***	***0.0308***			***21.44***
Common to Prob ID, Goal, & IQ	0.0005	0.0005		0.0005		0.35
Common to Prob ID, Solution, & IQ	-0.0010		-0.0010	-0.0010		-0.68
Common to Goal, Solution, & IQ		-0.0007	-0.0007		-0.0007	-0.47
Common to Prob ID, Goal, & Age	-0.0001	-0.0001			-0.0001	-0.09
Common to Prob ID, Solution, & Age	0.0028		0.0028		0.0028	1.98
Common to Goal, Solution, & Age		-0.0002	-0.0002		-0.0002	-0.13
Common to Prob ID, IQ, & Age	-0.0003			-0.0003	-0.0003	-0.21
Common to Goal, IQ, & Age		-0.0002		-0.0002	-0.0002	-0.12
Common to Solution, IQ, & Age			0.0003	0.0003	0.0003	0.18
Common to Prob ID, Goal, Solution, & IQ	-0.0007	-0.0007	-0.0007	-0.0007		-0.51
Common to Prob ID, Goal, Solution, & Age	0.0039	0.0039	0.0039		0.0039	2.72
Common to Prob ID, Goal, IQ, & Age	-0.0001	-0.0001		-0.0001	-0.0001	-0.05
Common to Prob ID, Solution, IQ, & Age	0.0001		0.0001	0.0001	0.0001	0.07
Common to Goal, Solution, IQ, & Age		0.0004	0.0004	0.0004	0.0004	0.29
Common to all five predictors	0.0002	0.0002	0.0002	0.0002	0.0002	0.17

Notes. A. Prob ID = Problem Identification; b. Goal = Goal Preference; c. Solution = Solution Preference; d. IQ = Full-Scale IQ measured with the KBIT-2; e. Age = Age at study visit; f. % Total refers to the percent of variance the combination of predictors, or each predictor uniquely, contributes to each social competency model; g. SSIS = Social Skills Improvement System Rating Scale standard score; Bolded values indicate which SPS component(s), individually or combined, contribute the most variance within each model.

Values in 2nd through 4th columns denote commonality coefficients, which represent the isolated, unique, and common variance each predictor (and combination of predictors) contributes to the dependent variable.

Supplemental Table 2. *SRS-2 Commonality Analyses with Age and IQ as Covariates*

**SRS-2[g] Predictors** (R^2=.15)	**Prob ID[a]**	**Goal[b]**	**Solution[c]**	**IQ[d]**	**Age[e]**	**%Total[f]**
Unique to Prob ID	***0.0471***					***31.57***
Unique to Goal		0.0017				1.15
Unique to Solution			0.0106			7.09
Unique to IQ				0.0142		9.50
Unique to Age					0.0014	0.92
Common to Prob ID & Goal	0.0025	0.0025				1.65
Common to Prob ID & Solution	0.0177		0.0177			11.84
Common to Goal & Solution		0.0179	0.0179			12.03
Common to Prob ID & IQ	0.0052			0.0052		3.50
Common to Goal & IQ		0.0010		0.0010		0.69
Common to Solution & IQ			-0.0044	-0.0044		-2.96
Common to Prob ID & Age	-0.0012				-0.0012	-0.80
Common to Goal & Age		0.0002			0.0002	0.10
Common to Solution & Age			-0.0003		-0.0003	-0.21
Common to IQ & Age				0.0006	0.0006	0.40
Common to Prob ID, Goal, & Solution	***0.0433***	***0.0433***	***0.0433***			***29.03***
Common to Prob ID, Goal, & IQ	0.0011	0.0011		0.0011		0.71
Common to Prob ID, Solution, & IQ	-0.0035		-0.0035	-0.0035		-2.35
Common to Goal, Solution, & IQ		-0.0028	-0.0028	-0.0028		-1.88
Common to Prob ID, Goal, & Age	-0.0001	-0.0001			-0.0001	-0.06
Common to Prob ID, Solution, & Age	0.0001		0.0001		0.0001	0.10
Common to Goal, Solution, & Age		0.0001	0.0001		0.0001	0.06
Common to Prob ID, IQ, & Age	-0.0004			-0.0004	-0.0004	-0.24
Common to Goal, IQ, & Age		0.0001		0.0001	0.0001	0.07
Common to Solution, IQ, & Age			-0.0001	-0.0001	-0.0001	-0.05
Common to Prob ID, Goal, Solution, & IQ	-0.0023	-0.0023	-0.0023	-0.0023		-1.57
Common to Prob ID, Goal, Solution, & Age	-0.0001	-0.0001	-0.0001		-0.0001	-0.08
Common to Prob ID, Goal, IQ, & Age	0.0000	0.0000		0.0000	0.0000	-0.02
Common to Prob ID, Solution, IQ, & Age	-0.0001		-0.0001	-0.0001	-0.0001	-0.08
Common to Goal, Solution, IQ, & Age		-0.0001	-0.0001	-0.0001	-0.0001	-0.09
Common to all five predictors	0.0000	0.0000	0.0000	0.0000	0.0000	-0.03

Notes. A. Prob ID = Problem Identification; b. Goal = Goal Preference; c. Solution = Solution Preference; d. IQ = Full-Scale IQ measured with the KBIT-2; e. Age = Age at study visit; f. % Total refers to the percent of variance the combination of predictors, or each predictor uniquely, contributes to each social competency model; g. SRS-2 = Social Responsiveness Scale, 2nd Edition, total *T*-score; Bolded values indicate which SPS component(s), individually or combined, contribute the most variance within each model.

Values in 2nd through 4th columns denote commonality coefficients, which represent the isolated, unique, and common variance each predictor (and combination of predictors) contributes to the dependent variable.

Supplementary Table 3. *ADOS-2 CSS Commonality Analyses with Age and IQ as Covariates*

**ADOS-2 CSS[g] Predictors** (R^2=.12)	**Prob ID[a]**	**Goal[b]**	**Solution[c]**	**IQ[d]**	**Age[e]**	**%Total[f]**
Unique to Prob ID	0.0152					12.54
Unique to Goal		0.0009				0.74
Unique to Solution			0.0045			3.68
Unique to IQ				***0.0484***		***40.05***
Unique to Age					0.0169	13.97
Common to Prob ID & Goal	0.0010	0.0010				0.80
Common to Prob ID & Solution	0.0064		0.0064			5.27
Common to Goal & Solution		0.0081	0.0081			6.69
Common to Prob ID & IQ	-0.0044			-0.0044		-3.66
Common to Goal & IQ		-0.0008		-0.0008		-0.66
Common to Solution & IQ			0.0095	0.0095		7.89
Common to Prob ID & Age	-0.0032				-0.0032	-2.64
Common to Goal & Age		0.0004			0.0004	0.34
Common to Solution & Age			-0.0007		-0.0007	-0.60
Common to IQ & Age				-0.0033	-0.0033	-2.69
Common to Prob ID, Goal, & Solution	0.0165	0.0165	0.0165			13.62
Common to Prob ID, Goal, & IQ	-0.0006	-0.0006		-0.0006		-0.48
Common to Prob ID, Solution, & IQ	0.0022		0.0022	0.0022		1.82
Common to Goal, Solution, & IQ		0.0043	0.0043	0.0043		3.53
Common to Prob ID, Goal, & Age	-0.0000	-0.0000			-0.0000	-0.02
Common to Prob ID, Solution, & Age	-0.0010		-0.0010		-0.0010	-0.85
Common to Goal, Solution, & Age		0.0002	0.0002		0.0002	0.18
Common to Prob ID, IQ, & Age	0.0009			0.0009	0.0009	0.72
Common to Goal, IQ, & Age		-0.0002		-0.0002	-0.0002	-0.20
Common to Solution, IQ, & Age			-0.0008	-0.0008	-0.0008	-0.68
Common to Prob ID, Goal, Solution, & IQ	0.0021	0.0021	0.0021	0.0021		1.75
Common to Prob ID, Goal, Solution, & Age	-0.0015	-0.0015	-0.0015		-0.0015	-1.22
Common to Prob ID, Goal, IQ, & Age	0.0001	0.0001		0.0001	0.0001	0.06
Common to Prob ID, Solution, IQ, & Age	-0.0001		-0.0001	-0.0001	-0.0001	-0.05
Common to Goal, Solution, IQ, & Age		0.0001	0.0001	0.0001	0.0001	0.11
Common to all five predictors	0.0000	0.0000	0.0000	0.0000	0.0000	-0.03

Notes. A. Prob ID = Problem Identification; b. Goal = Goal Preference; c. Solution = Solution Preference; d. IQ = Full-Scale IQ measured with the KBIT-2; e. Age = Age at study visit; f. % Total refers to the percent of variance the combination of predictors, or each predictor uniquely, contributes to each social competency model; g. ADOS-2 CSS = Autism Diagnostic Observation Schedule, 2nd Edition, Comparison Severity Score; Bolded values indicate which SPS component(s), individually or combined, contribute the most variance within each model.

Values in 2nd through 4th columns denote commonality coefficients, which represent the isolated, unique, and common variance each predictor (and combination of predictors) contributes to the dependent variable.
